# Exploratory Temporal Dynamics of Heart-Rate Variability During a Prolonged 2-Back Task Under Nap-Deprivation Conditions

**DOI:** 10.3390/s26196099

**Published:** 2026-09-25

**Authors:** Di Meng, Luyao Yu, Tianjiao Min, Jing Zhang, Liufeng Zhu, Hengcong Gong, Yifei Feng, Ying He

**Affiliations:** 1 Naval Medical Center, Naval Medical University, Shanghai 200433, China; 15062829514@163.com (D.M.); y777ly@163.com (L.Y.); mtj0528@163.com (T.M.); 18754881136@163.com (J.Z.); z18451308865@163.com (L.Z.); gonghengcong@gmail.com (H.G.); 2 School of Health Science and Engineering, University of Shanghai for Science and Technology, Shanghai 200093, China

**Keywords:** heart-rate variability, ballistocardiogram, prolonged cognitive task, 2-back, mental-fatigue induction, time-on-task, fiber-optic sensing

## Abstract

Mental fatigue is commonly studied using prolonged cognitive tasks, but physiological changes observed in single-group paradigms may also reflect time-on-task, circadian variation, nap deprivation, posture, and cognitive load. This exploratory study characterized continuous heart-rate variability (HRV) dynamics during a 60-min 2-back task performed in the habitual afternoon nap period. Twenty-six healthy graduate students completed pre- and post-task assessments using the mental-fatigue subscale of the Fatigue Self-Assessment Scale (FSAS) and a simple reaction-time test; 22 participants were included in the HRV analysis after four recordings were excluded because insufficient compliance with the required seated protocol resulted in inadequate valid data. Ballistocardiogram signals were acquired using a 1000-Hz fiber-optic sensing cushion. Nineteen HRV features were estimated in 5-min windows with a 1-min step, and temporal dynamics were described using baseline-relative change (Delta) and log change rate (LCR). The FSAS mental-fatigue score increased from 18.99±17.09 to 46.88±24.89 (mean change 27.88, 95% CI 19.34–36.43; paired t(25)=6.72, p<0.001; Cohen’s $d_{z}=1.32$), and reaction time increased from 371.63±37.74 to 407.62±66.70 ms (mean change 35.98 ms, 95% CI 21.17–50.80; t(25)=5.00, p<0.001; $d_{z}=0.98$). A BCG-derived respiratory surrogate showed a phase effect for relative respiratory amplitude but not respiratory rate. Across the 19 HRV features included in the primary analysis, several nominal phase effects were observed, but none remained significant after Benjamini–Hochberg false-discovery-rate correction. Exploratory ranking and dynamic time-warping clustering indicated heterogeneous feature trajectories, although the fixed five-cluster solution was not the best-separated solution and participant-level reproducibility was limited. These findings support the feasibility of unobtrusive BCG for describing continuous physiological variation during prolonged cognitive engagement, but they do not establish fatigue-specific HRV phases, diagnostic markers, or individual-level monitoring performance.

## 1. Introduction

Mental fatigue is a psychophysiological state associated with prolonged cognitive effort and is commonly accompanied by reduced alertness, slower responses, and impaired task performance [1,2,3]. Continuous assessment is relevant to occupations requiring sustained attention, including driving, vigilance, healthcare, and shift work [4,5]. However, mental fatigue is not directly observable through a single physiological variable, and changes recorded during laboratory induction tasks may reflect several overlapping influences, including cognitive load, time-on-task, boredom, sleepiness, circadian timing, and posture.

Heart-rate variability (HRV) describes beat-to-beat variation in cardiac intervals and is influenced by multiple autonomic and non-autonomic processes [6,7,8,9,10]. Sympathetic modulation broadly refers to neural influences that support cardiovascular activation, whereas parasympathetic modulation includes vagally mediated influences that can slow and dynamically regulate cardiac timing. Individual HRV indices are indirect and non-specific: for example, RMSSD, pNN50, HF, and SD1 are influenced by parasympathetic activity but also by respiration, heart rate, preprocessing choices, and signal quality [11,12,13,14,15]; LF/HF should not be interpreted as a direct measure of sympathovagal balance [7,8,11,12,15].

Prior work has reported HRV changes during sustained cognitive tasks and mental-fatigue paradigms [16,17,18,19,20,21,22]. Most studies emphasize pre–post comparisons, in which measurements obtained before a prolonged task are compared with those obtained immediately after the task, or assess only a limited number of intermediate time points. Such approaches may miss transient within-task changes and between-feature heterogeneity. Continuous ballistocardiography (BCG), which records small body movements associated with cardiac ejection and blood movement, provides a potential unobtrusive route for long-duration cardiac-interval monitoring [23,24,25]. In BCG recordings, successive J-wave peaks can be detected, and the intervals between adjacent J peaks (J–J, JJ intervals) can serve as mechanical surrogates for electrocardiogram (ECG) R–R (RR) intervals. After artifact rejection and interval-quality control, HRV indices can be calculated from the resulting JJ-interval series [25,26]. Because mechanical peak detection is sensitive to motion, posture, and signal quality, missed, spurious, or displaced J peaks can introduce JJ-interval estimation errors that subsequently propagate into HRV estimates, particularly analyses based on changes between consecutive analysis windows.

To avoid ambiguity in the terminology used below, a window refers to a 5-min segment of BCG-derived HRV data, and the step refers to the 1-min temporal advance between consecutive windows. A phase refers to a broader descriptive time block containing multiple windows: Early (0–10 min), Change (10–20 min), Mid (20–40 min), and Late (40–60 min). The term interval is used specifically for beat-to-beat cardiac intervals (JJ or RR intervals), unless otherwise stated. Baseline-relative change (Delta) describes cumulative deviation from the participant-specific resting baseline, whereas log change rate (LCR) describes local proportional change between adjacent analysis windows.

The methodological contribution of the present study lies in integrating continuous fiber-optic BCG acquisition with time-resolved HRV analysis rather than in proposing a new individual HRV metric. Delta and LCR provide complementary temporal descriptions: Delta quantifies how far each HRV feature deviates from the participant-specific resting baseline over the course of the task, whereas LCR quantifies short-term proportional changes between adjacent windows. Participant-level summaries were then calculated within the broader descriptive phases before repeated-measures statistical testing, thereby avoiding treatment of highly overlapping windows as independent observations. Exploratory ranking and trajectory clustering were subsequently used only to describe between-feature temporal heterogeneity and were not used for confirmatory feature selection.

Because all participants completed the same 2-back task during their habitual nap period and no control condition was available, the study was framed as an exploratory description of HRV during a prolonged cognitive task under nap-deprivation conditions. The objectives were to: (1) verify group-level fatigue manipulation using the pre–post FSAS mental-fatigue subscale and simple reaction time; (2) characterize continuous HRV trajectories; (3) test phase-related differences across all 19 HRV features included in the primary analysis with false-discovery-rate control; and (4) evaluate the sensitivity of descriptive rankings and trajectory clusters without treating them as validated fatigue biomarkers or participant subtypes.

## 2. Materials and Methods

### 2.1. Participants, Recruitment, and Analysis Samples

Twenty-six healthy graduate students (16 women and 10 men) aged 23–31 years were enrolled. The reported mean age was 25±2 years, and the mean body mass index was 20.97±2.00 kg/m^2^. Participants were eligible if they were 18–35 years old, maintained a self-reported regular sleep–wake schedule, reported sufficient sleep on the preceding night, and refrained from smoking, alcohol, coffee, tea, and energy drinks on the experimental day. Exclusion criteria included major organic disease involving the liver, kidney, heart, brain, or lungs; severe mental disorder; pregnancy or lactation; and substance or alcohol dependence.

Habitual napping was operationalized as self-reported regular use of the institutional midday rest period (12:30–14:00). Nap deprivation was defined as completing the experimental session during this usual nap period without sleeping. These eligibility and compliance variables were verified by self-report rather than actigraphy, biochemical testing, or long-term sleep monitoring.

All 26 participants completed the pre–post fatigue-manipulation assessments. Four participants were excluded from the HRV analysis because insufficient compliance with the required prolonged seated protocol (for example, standing or major movement) resulted in inadequate task duration, valid RR intervals, or valid analysis windows. Therefore, 22 participants contributed to the HRV, respiration-surrogate, ranking, and clustering analyses. No a priori sample-size calculation was performed; the study is exploratory and hypothesis-generating. Race or ethnicity data were not available for the present analysis, see Table 1.

### 2.2. Experimental Protocol and Fatigue-Manipulation Checks

Each participant completed one experimental session beginning at approximately 13:00. A 2-back working-memory task was used to provide sustained cognitive load. Letters were presented sequentially, and participants indicated whether the current letter matched the letter shown two positions earlier. A brief practice session was completed before the formal task. The formal task lasted 60 min, with each stimulus displayed for 500 ms at a fixed presentation pace.

A schematic illustration of the 2-back matching rule is shown in Figure 1.

Before the task, participants completed the mental-fatigue subscale of the Fatigue Self-Assessment Scale (FSAS) and a simple reaction-time test, followed by a 10-min seated resting BCG recording. The mental-fatigue subscale contains four items and can be administered independently. Each item is rated on a five-point scale from 0 (“none or occasionally”) to 4 (“almost all the time”). The four item scores are summed, divided by the maximum possible score of 16, and multiplied by 100 to obtain a standardized mental-fatigue score ranging from 0 to 100, with higher scores indicating greater subjective mental fatigue. The FSAS mental-fatigue subscale and the reaction-time test were repeated immediately after the 60-min task. In the present study, the FSAS mental-fatigue score and simple reaction time were analyzed as separate pre–post manipulation-check outcomes. Minute-level 2-back accuracy, omissions, false responses, and reaction-time trajectories were not retained in an analyzable form and therefore could not be reported. Three duplicate questionnaire entries were identified; for consistency, the second complete record was retained for each duplicate.

BCG signals were recorded using a Mach–Zehnder interferometer (MZI) fiber-optic sensing cushion at 1000 Hz. Participants remained seated during the 10-min baseline and the subsequent 60-min task.

The overall experimental sequence and BCG acquisition protocol are summarized in Figure 2.

The same MZI-BCG (custom-built system) system was previously validated against simultaneous electrocardiogram (ECG) recordings [26]. BCG-derived heart-rate estimates showed high agreement with ECG-derived heart-rate estimates (Pearson’s R=0.9986; RMSE = 0.7732 bpm), and most measurements were within the 95% limits of agreement in Bland–Altman analysis. In the present study, preliminary comparisons using a Polar H10 ECG sensor (Polar Electro Oy, Kempele, Finland) and an HKH-11C respiratory sensor (Hefei Huake Electronic Technology Research Institute, Hefei, China) yielded maximum correlations of 0.9289 and 0.9053 for heart rate and the respiratory-sensor output, respectively. All analyses were performed in MATLAB R2022b (MathWorks, Natick, MA, USA). These results support the measurement feasibility of the device, although complete agreement analysis of cardiac intervals and HRV indices during the 2-back task was not available and is acknowledged as a limitation.

### 2.3. Signal Processing, Quality Control, and HRV Feature Extraction

The MZI-BCG platform used in this study has previously been evaluated against simultaneous ECG recordings. BCG-derived JJ intervals showed strong agreement with ECG-derived RR intervals (R=0.9986, RMSE = 0.7732 bpm), with most observations falling within the 95% Bland–Altman limits of agreement [26]. This supports device-level validity but does not replace task-specific validation of the present recordings.

A complete 10-min baseline and at least 55 min of usable task data were required. Signals were filtered using a third-order zero-phase Butterworth band-pass filter (1–5 Hz), followed by first-difference enhancement. Peaks were detected using MATLAB findpeaks with a minimum distance of 0.45 s and a height threshold of 0.60 times the standard deviation of the first-difference signal. Consecutive peaks were converted to NN-like intervals.

Intervals outside 300–2000 ms or differing by more than 20% from the local 11-interval median were rejected. Up to two consecutive missing intervals were interpolated. Each 5-min window required at least 150 valid intervals and 80% temporal coverage. Participants were required to provide at least 40 valid windows.

Nineteen HRV features were calculated: MeanNN, MedianNN, SDNN, RMSSD, SDSD, pNN50, CVNN, CVSD, HR, VLF, LF, HF, LF/HF, TP, SD1, SD2, SD1/SD2, ApEn, and SampEn. Frequency-domain analysis used 4-Hz piecewise cubic Hermite interpolating polynomial (PCHIP) interpolation, detrending, and Welch spectral estimation with a 256-sample Hamming window and 50% overlap. Frequency bands were VLF (0.0033–0.04 Hz), LF (0.04–0.15 Hz), and HF (0.15–0.40 Hz). ApEn and SampEn used m=2 and r=0.20 times the within-window standard deviation. All analyses were performed in MATLAB R2022b.

### 2.4. Respiratory-Surrogate Analysis

For the task-wide analysis, a respiratory surrogate was extracted from the low-frequency BCG component because a complete independent respiratory-sensor series was not available for all participants. The signal was repaired for finite-value gaps, robustly clipped for extreme artifacts, resampled to 10 Hz, and filtered using a second-order zero-phase 0.10–0.40 Hz band-pass filter. Respiratory rate and relative root-mean-square amplitude were summarized by phase.

The relative amplitude is an uncalibrated BCG-derived quantity and is not a measure of absolute breathing depth. Respiratory results were used only as auxiliary evidence of task-related physiological change and as a reminder that respiration may confound HF and LF/HF. No mechanistic correlation or covariate-adjusted analysis between respiration and HRV was performed.

### 2.5. Temporal Metrics and Analysis Windows

HRV features were computed in 300-s windows with a 60-s step, corresponding to 80% overlap. A complete 60-min task produced a theoretical maximum of 56 windows centered at 2.5–57.5 min; shorter retained recordings contributed all complete windows within their available duration. The 10-min resting segment was used to calculate participant-specific baseline feature values.

For feature *F* in task window *i*, the baseline-relative change was

(1) $$\Delta_{i}=\frac{F_{i}-F_{\mathrm{base}}}{F_{\mathrm{base}}},$$

which is a dimensionless ratio representing cumulative deviation from baseline. The log change rate was

(2) $${\mathrm{LCR}}_{i}=\ln\left(F_{i}+\varepsilon \right)-\ln\left(F_{i-1}+\varepsilon \right),\;\varepsilon =10^{-6},$$

which represented local proportional change between adjacent windows and was the primary dynamic metric [27]. The adjacent-window percentage change was

(3) $$\mathrm{CR}{\%}_{i}=100\frac{F_{i}-F_{i-1}}{F_{i-1}},$$

and was retained only as an auxiliary intuitive measure [28]. Phase-level change intensity was defined as the participant-level median of |LCR|, and zero-crossing count described directional alternation, see Table 2.

### 2.6. Descriptive Phase Windows and Sensitivity Analyses

The task was summarized using four descriptive phases: Early (0–10 min), Change (10–20 min), Mid (20–40 min), and Late (40–60 min). These labels were used solely for descriptive presentation and were not treated as validated physiological transition points. Because the four phases differed in duration, participant-level medians were calculated within each phase before repeated-measures testing so that phases containing more overlapping windows did not contribute proportionally more observations to the statistical analysis.

Sensitivity analyses used four equal 15-min phases and three equal 20-min phases. Additional analyses varied window length and step size (3-min/1-min, 5-min/1-min, 5-min/2-min, and 5-min/5-min) and ε ($10^{-8}$, $10^{-6}$, and $10^{-4}$). These analyses were used to evaluate dependence on analytical choices rather than to select the setting that produced the smallest *p* values.

### 2.7. Exploratory Dynamic-Response Ranking and Trajectory Clustering

After the primary inferential analysis of all 19 HRV features, an exploratory dynamic-response ranking was computed from four min–max normalized components: absolute global Delta slope (weight 0.25), maximum phase difference in Delta (0.35), mean participant-level median |LCR| (0.25), and mean LCR zero-crossing count (0.15). The score was scaled to 0–10, following the general principle of constructing weighted composite indicators [29]. It was not interpreted as a validated sensitivity measure and was not used to select outcomes for confirmatory testing. Ranking uncertainty was evaluated using 5000 randomly sampled weight vectors, reporting rank ranges and the probability of appearing in the top five.

For clustering, group-mean LCR trajectories were aligned to a common time grid, missing late time points were retained as missing rather than imputed beyond the available recording, and each feature trajectory was z-standardized across time. Pairwise DTW distances were clustered using average linkage [30,31]. Candidate cluster solutions with K=3, 4, and 5 were compared using silhouette coefficients. A fixed K=5 partition was additionally retained as an exploratory candidate solution to examine the interpretability and stability of a five-cluster representation. Cluster stability was examined using 1000 participant-level bootstrap resamples [32]. Clustering was applied to HRV feature trajectories, not to participants. Participant-level reproducibility was separately assessed using the Spearman correlation between each participant trajectory and the corresponding leave-one-participant-out group trajectory.

### 2.8. Statistical Analysis

Pre–post FSAS mental-fatigue scores and reaction time were evaluated using paired *t* tests, 95% confidence intervals for the mean change, Wilcoxon signed-rank tests, and Cohen’s $d_{z}$. The respiratory-surrogate metrics were assessed descriptively using Friedman tests with Benjamini–Hochberg correction across the two respiratory outcomes.

For the primary HRV analysis, each of the 19 HRV features was tested using a Friedman repeated-measures test on participant-level phase median |LCR|. Friedman chi-square, exact *p* value, and Kendall’s *W* were reported. Benjamini–Hochberg false-discovery-rate (FDR) correction was applied across the 19 HRV outcomes. Holm-adjusted Wilcoxon paired comparisons with bootstrap 95% confidence intervals for the median paired difference were planned only for features that remained significant after FDR correction. Delta-trajectory figures display group means with *t*-based 95% confidence intervals and are explicitly labeled as ratios rather than percentages. All analyses were performed using MATLAB R2022b (MathWorks, Natick, MA, USA).

## 3. Results

### 3.1. Participant Flow and Fatigue-Manipulation Checks

All 26 participants completed the pre–post manipulation assessments. Four recordings were excluded from HRV analysis because inadequate compliance with the prolonged seated protocol resulted in insufficient valid physiological data; 22 participants remained for HRV analysis. Among included recordings, the usable task duration ranged from 58.93 to 60.00 min, and participants contributed a median of 56 valid HRV windows (range 47–56).

FSAS mental-fatigue scores increased from 18.99±17.09 to 46.88±24.89. The mean increase was 27.88 points (95% CI 19.34–36.43), t(25)=6.72, p<0.001, Cohen’s $d_{z}=1.32$; the Wilcoxon test was also significant (W=0, p<0.001). Simple reaction time increased from 371.63±37.74 to 407.62±66.70 ms, with a mean increase of 35.98 ms (95% CI 21.17–50.80), t(25)=5.00, p<0.001, $d_{z}=0.98$; the Wilcoxon test was significant (W=11, p<0.001). These results support a group-level increase in subjective fatigue and behavioral slowing after the protocol, but they do not isolate fatigue from time-on-task, nap deprivation, circadian timing, or other task-related factors, see Table 3.

### 3.2. Auxiliary Respiratory-Surrogate Results

Respiratory rate did not differ across the four descriptive task phases (Friedman $\chi^{2}=3.482$, p=0.323, FDR-adjusted p=0.323, W=0.053). Relative respiratory amplitude showed a phase effect ($\chi^{2}=13.745$, p=0.0033, FDR-adjusted p=0.0065, W=0.208). The phase medians were highest in the Late phase, but the amplitude was uncalibrated and highly variable. This result was treated as supplementary physiological evidence of task-related change and as evidence that respiration remained a plausible confounder, rather than as proof of a specific autonomic mechanism, see Table 4.

### 3.3. Descriptive HRV Trajectory Heterogeneity

The all-feature heatmap showed heterogeneous temporal variation in median |LCR| across features and time (Figure 3). Frequency-domain features generally displayed larger absolute LCR values than interval-location features such as MeanNN and MedianNN. The figure is descriptive: visual variation alone does not establish statistically distinct physiological phases or fatigue-specific mechanisms.

### 3.4. Phase Comparisons Across All 19 HRV Features

Several features showed nominal raw *p* values below 0.05, including CVSD, RMSSD, SDSD, SD1, VLF, and pNN50. However, none of the 19 features remained significant after Benjamini–Hochberg FDR correction. The lowest adjusted *p* value was 0.0596 for CVSD, RMSSD, SDSD, and SD1. Therefore, no Holm-adjusted pairwise post-hoc tests were performed. Figure 4 shows the eight features with the smallest raw *p* values for visualization; inference was based on the full 19-feature analysis in Table 5 and Supplementary Table S1.

### 3.5. Exploratory Dynamic-Response Ranking

The reference weighting placed pNN50, LF, VLF, HF, TP, and LF/HF at the top of the dataset-specific exploratory ranking. Random-weight analysis showed that LF and VLF most consistently appeared in the top five, whereas the position of pNN50, TP, and LF/HF was more weight-dependent. Because the ranking components were derived from the same data and were not externally validated, the ranking was not interpreted as evidence that one feature is a superior fatigue marker; see Table 6 and Figure 5.

### 3.6. DTW Clustering and Participant-Level Reproducibility

The mean silhouette coefficient was 0.380 for K=3, 0.291 for K=4, and 0.282 for the fixed K=5 solution. Therefore, K=5 was not the best-separated data-driven solution and is presented only as an exploratory candidate partition rather than as the optimal cluster structure. The fixed K=5 cut produced one large cluster containing 12 mathematically and physiologically related variability and spectral features, two single-feature clusters (HR and LF/HF), one MeanNN/MedianNN cluster, and one nonlinear-feature cluster (ApEn, SD1/SD2, and SampEn). Feature-level bootstrap stability values are displayed in Figure 6 and summarized in Table 7, but they should be interpreted together with the modest silhouette coefficients and the fixed cluster count.

Participant-level reproducibility was limited. Across the 19 features, median participant-to-leave-one-out-group Spearman correlations ranged from −0.091 to 0.115, and the proportion of participants with correlations of at least 0.30 was generally 0–9.1%. Thus, group-average feature trajectories should not be interpreted as reproducible participant subtypes. The close clustering of RMSSD, SDSD, and SD1 is expected in part because of their mathematical relationships and does not provide three independent physiological confirmations.

### 3.7. Sensitivity Analyses

Alternative phase segmentation did not change the inferential conclusion: no HRV feature was FDR significant under either four equal 15-min phases or three equal 20-min phases. The Kendall-*W* ranking across features correlated with the primary analysis at ρ=0.772 and ρ=0.830, respectively.

The results were invariant across ε values from $10^{-8}$ to $10^{-4}$ within each window–step configuration, but they were materially affected by window length and step size. The 3-min/1-min analysis yielded one FDR-significant feature, the primary 5-min/1-min analysis yielded none, the 5-min/2-min analysis yielded none, and the non-overlapping 5-min/5-min analysis yielded 11. These differences demonstrate that the inferential results depend on temporal aggregation and serial overlap. The 5-min/1-min configuration was therefore retained as the reference configuration for the primary analysis, while the alternative settings are reported transparently as sensitivity analyses, see Table 8.

## 4. Discussion

### 4.1. Manipulation Evidence and Scope of Inference

The large pre–post increase in the FSAS mental-fatigue score and the moderate-to-large behavioral slowing provide direct group-level evidence that the experimental protocol increased subjective fatigue and impaired simple response speed. Together, these subjective and behavioral measures provide complementary group-level manipulation checks indicating increased fatigue and behavioral slowing after the experimental protocol. The BCG-derived respiratory amplitude also varied over time, providing supplementary evidence of physiological change during the task.

Nevertheless, the single-group design cannot separate mental fatigue from time-on-task, nap deprivation, circadian variation, cognitive load, boredom, or prolonged sitting. The revised title, objectives, and conclusions therefore describe condition-specific responses during a prolonged 2-back task rather than a causal trajectory of mental-fatigue progression. The manipulation checks demonstrate that fatigue increased after the protocol, but they do not prove that every within-task HRV fluctuation was caused specifically by fatigue.

### 4.2. HRV Temporal Heterogeneity Without Multiplicity-Corrected Phase Effects

The descriptive heatmap showed substantial between-feature temporal heterogeneity. However, after testing all 19 HRV features and controlling the false discovery rate, no phase effect remained statistically significant. This multiplicity-controlled analysis supports a more conservative interpretation than one based on nominal *p* values or a limited subset of HRV features. The data support temporal variation and heterogeneity at the descriptive level, but not a validated four-stage HRV model.

CVSD, RMSSD, SDSD, SD1, VLF, and pNN50 showed the smallest raw *p* values and small-to-moderate Kendall-*W* values, which may motivate future preregistered studies. Their adjusted *p* values, however, exceeded 0.05. The absence of corrected significance may reflect limited power, inter-individual variability, overlapping-window dependence, measurement noise, or genuinely weak phase effects. It should not be reframed as confirmation based on nominal *p* values.

### 4.3. Interpretation of Exploratory Ranking and Clustering

The exploratory ranking identified frequency-domain powers and pNN50 as dynamically active under the chosen weighting scheme. Random-weight analysis showed that some top positions were relatively stable, but others changed substantially. The ranking is therefore useful for descriptive prioritization and hypothesis generation, not for declaring superior fatigue sensitivity.

DTW clustering provided a compact description of trajectory similarity. The K=3 solution had a higher silhouette coefficient than K=5, and the fixed K=5 solution contained two single-feature clusters and one dominant 12-feature cluster. The clustering should therefore be interpreted as an exploratory partition rather than confirmation of five distinct physiological patterns. Rule-based morphology labels and DTW cluster membership were kept separate. Stable or reversing trajectories could hypothetically arise from changes in autonomic regulation, but they could also occur during other forms of sustained cognitive stress. Mechanistic labels such as vagal withdrawal, compensation, or adaptation are not warranted without controlled respiration and additional physiological measurements.

### 4.4. Respiration and Physiological Interpretation

Respiration is especially relevant to HF and LF/HF. The present BCG-derived surrogate showed a phase effect in relative amplitude, while respiratory rate did not. Because the amplitude was uncalibrated and not incorporated as a covariate, respiratory changes remain a plausible alternative explanation for part of the frequency-domain variation.

Accordingly, HF, RMSSD, pNN50, and SD1 are described as indices influenced by parasympathetic modulation rather than direct measures of vagal activity. LF/HF is retained as a descriptive spectral ratio only. The revised manuscript does not interpret spectral changes as direct evidence of sympathovagal balance, active compensation, or autonomic readjustment.

### 4.5. Dependence on Analytical Choices

The epsilon sensitivity analysis indicated that the tested logarithmic offset was not a major determinant of results. In contrast, window length and step size materially affected the number of FDR-significant outcomes and the ranking of effect sizes. Highly overlapping windows generate serial dependence, whereas non-overlapping windows reduce temporal resolution and change within-phase precision. These findings argue against claiming universal robustness of LCR and support explicit reporting of the selected temporal aggregation.

The 5-min window with a 1-min step was used as the primary analysis configuration because it is compatible with conventional short-term HRV analysis while allowing time-resolved tracking across the 60-min task. Because the study protocol and analysis plan were not preregistered, this configuration should be regarded as the primary analytical setting used in the present study rather than as a preregistered specification. VLF estimates from 5-min segments remain exploratory, and the large Delta ratios of features with low baseline values should be interpreted cautiously.

### 4.6. Engineering Implications, Generalizability, and Limitations

The fiber-optic cushion demonstrates an engineering route for unobtrusive, long-duration BCG acquisition. A future real-time system could compute quality-controlled cardiac intervals and rolling HRV descriptors, but the present study did not develop a classifier, calibration model, fatigue threshold, sensitivity, specificity, or external validation. Practical deployment would additionally require robust motion-artifact handling, posture monitoring, independent respiration, device-agreement validation, and prospective testing in operational settings.

The sample was small, homogeneous, and restricted to young graduate students. Four of 26 recordings were excluded because of inadequate compliance and valid signal availability, reducing the HRV sample to 22. No a priori power analysis was performed. Responses may differ in older adults and in drivers, pilots, healthcare workers, industrial operators, or shift workers. The nap-deprivation condition may amplify sleepiness and may not generalize to fully rested participants.

Additional limitations include the lack of a rest or non-fatiguing control condition, the absence of analyzable minute-level 2-back performance, self-reported sleep and stimulant compliance, use of a BCG-derived rather than independently calibrated task-wide respiratory signal, incomplete device-agreement statistics, analysis of one experimental dataset, and limited participant-level trajectory reproducibility. Future studies should preregister outcomes and analysis settings, include control conditions, retain continuous behavioral performance, use independent respiratory monitoring, and validate results across tasks, populations, devices, and external datasets.

## 5. Conclusions

The prolonged 2-back protocol produced substantial increases in subjective fatigue and simple reaction time. Continuous fiber-optic BCG captured heterogeneous HRV trajectories, but no HRV feature showed a statistically significant four-phase effect after FDR correction across 19 outcomes. Exploratory ranking and DTW clustering summarized feature-level temporal variation but did not establish an optimal five-pattern taxonomy, participant fatigue subtypes, diagnostic markers, or real-time detection performance. The results should be interpreted as condition-specific, hypothesis-generating evidence that unobtrusive BCG can describe continuous physiological variation during prolonged cognitive engagement. Replication with larger and more diverse samples, control conditions, continuous behavioral data, independent respiration, and external validation is required.

## Figures and Tables

**Figure 1:**
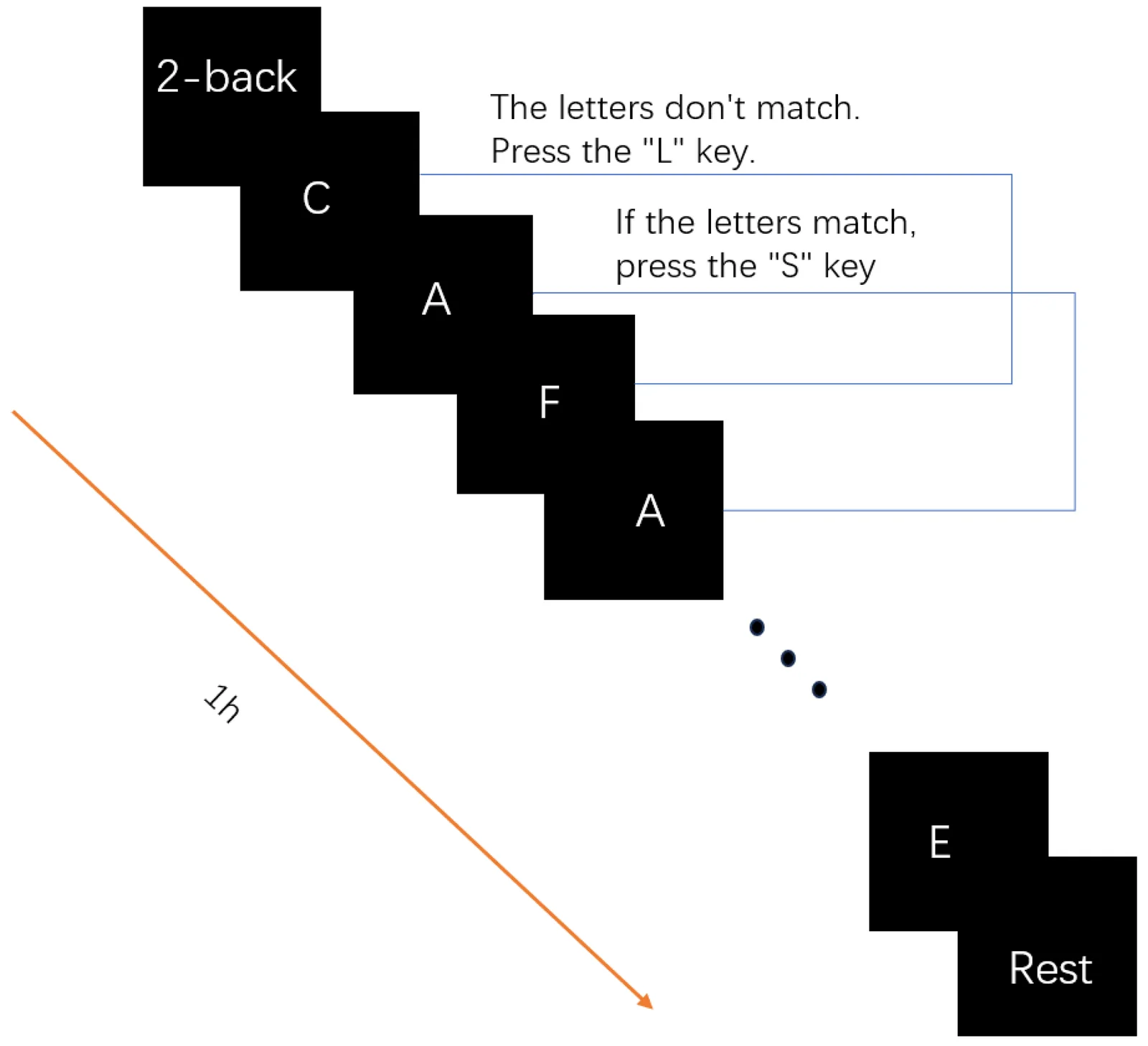
Schematic illustration of the 2-back working-memory task. Participants judged whether the current letter matched the letter presented two positions earlier. The figure is provided to clarify the task rule and sequence. (Note: The three dots denote omitted repeated experimental procedures).

**Figure 2:**
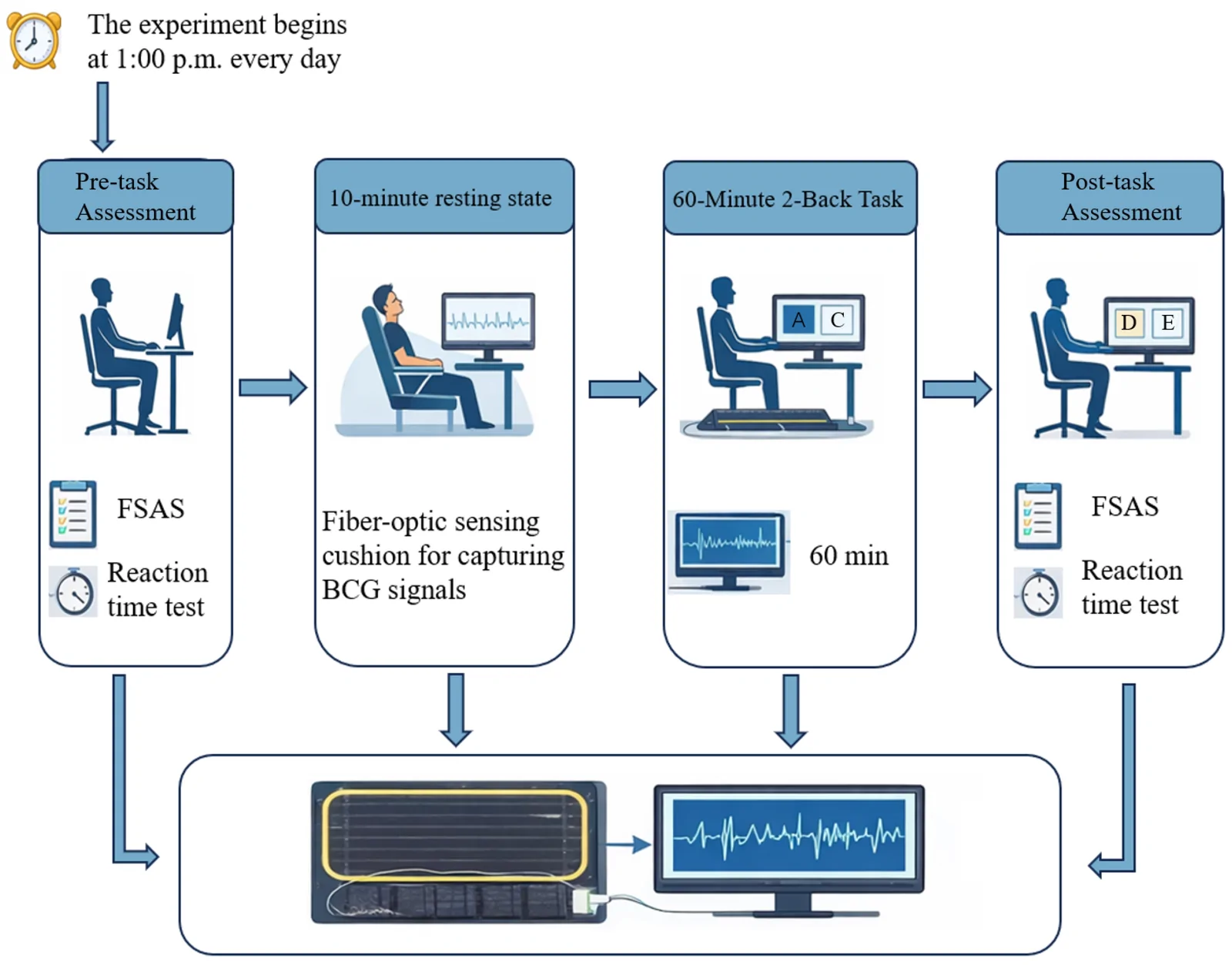
Experimental protocol and BCG data acquisition. The session began at approximately 13:00 and included a pre-task assessment (FSAS mental-fatigue subscale and simple reaction-time test), a 10-min seated resting BCG recording, a 60-min 2-back task with continuous BCG acquisition, and an immediate post-task assessment using the same FSAS subscale and reaction-time test.

**Figure 3:**
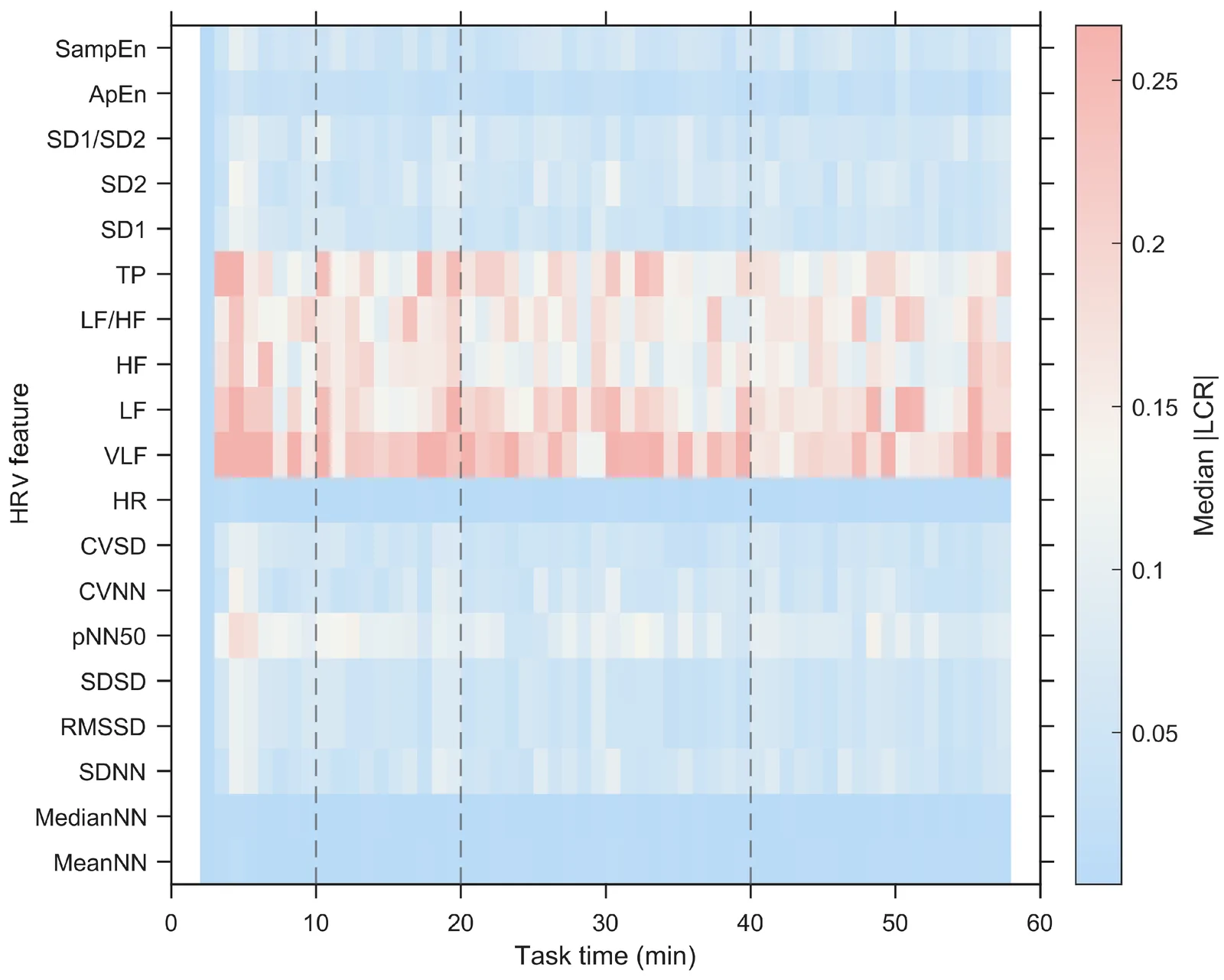
Group-level median absolute log change rate (|LCR|) across task time for the 19 HRV features. Vertical dashed lines indicate the descriptive phase boundaries. The color scale represents a dimensionless log-change magnitude.

**Figure 4:**
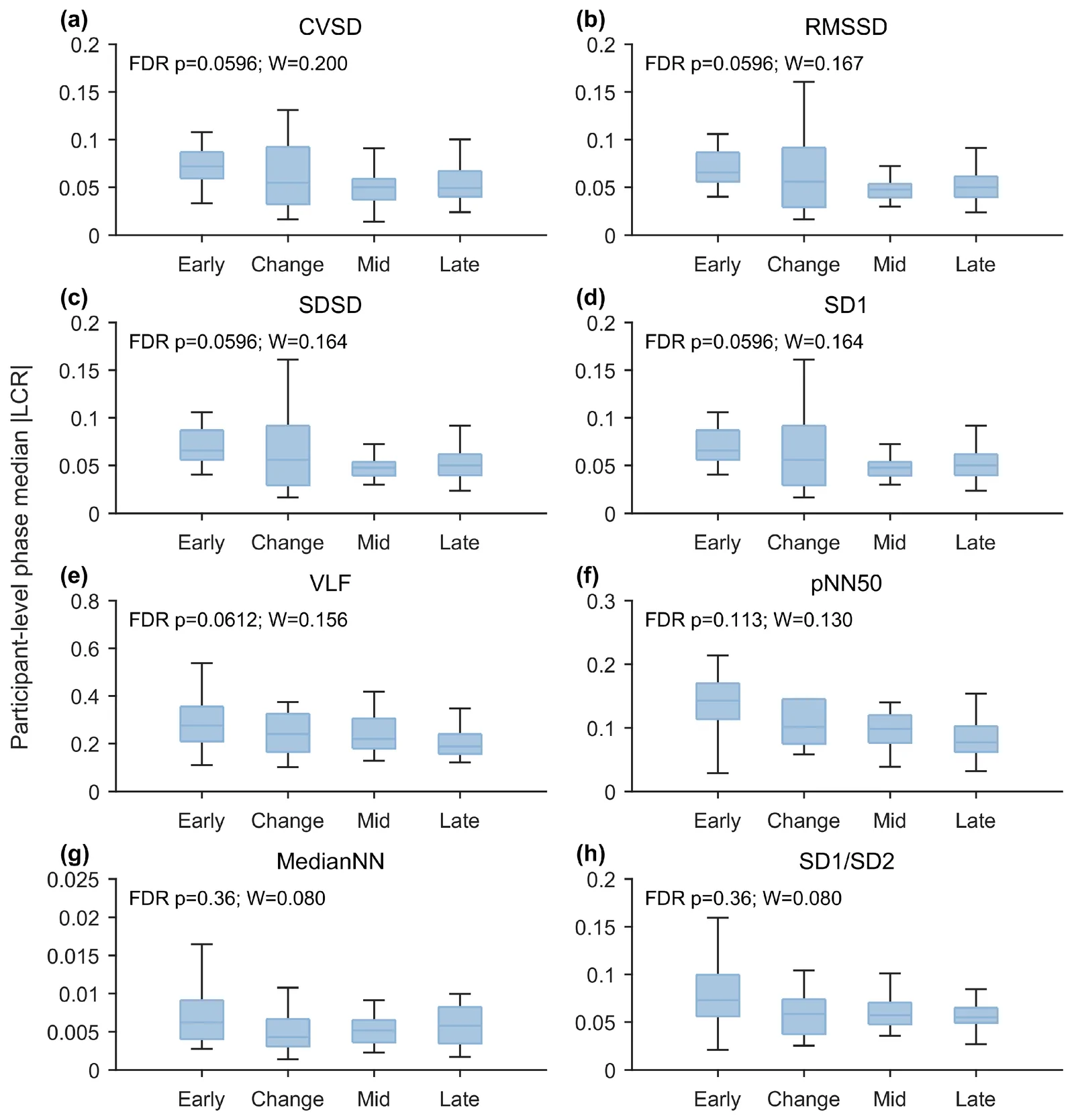
Participant-level phase median |LCR| for the eight features with the smallest raw *p* values.Panels show (**a**) CVSD, (**b**) RMSSD, (**c**) SDSD, (**d**) SD1, (**e**) VLF, (**f**) pNN50, (**g**) MedianNN, and (**h**) SD1/SD2.Boxes are descriptive; FDR-adjusted *p* values and Kendall’s *W* are shown in each panel. No feature met the FDR significance criterion.

**Figure 5:**
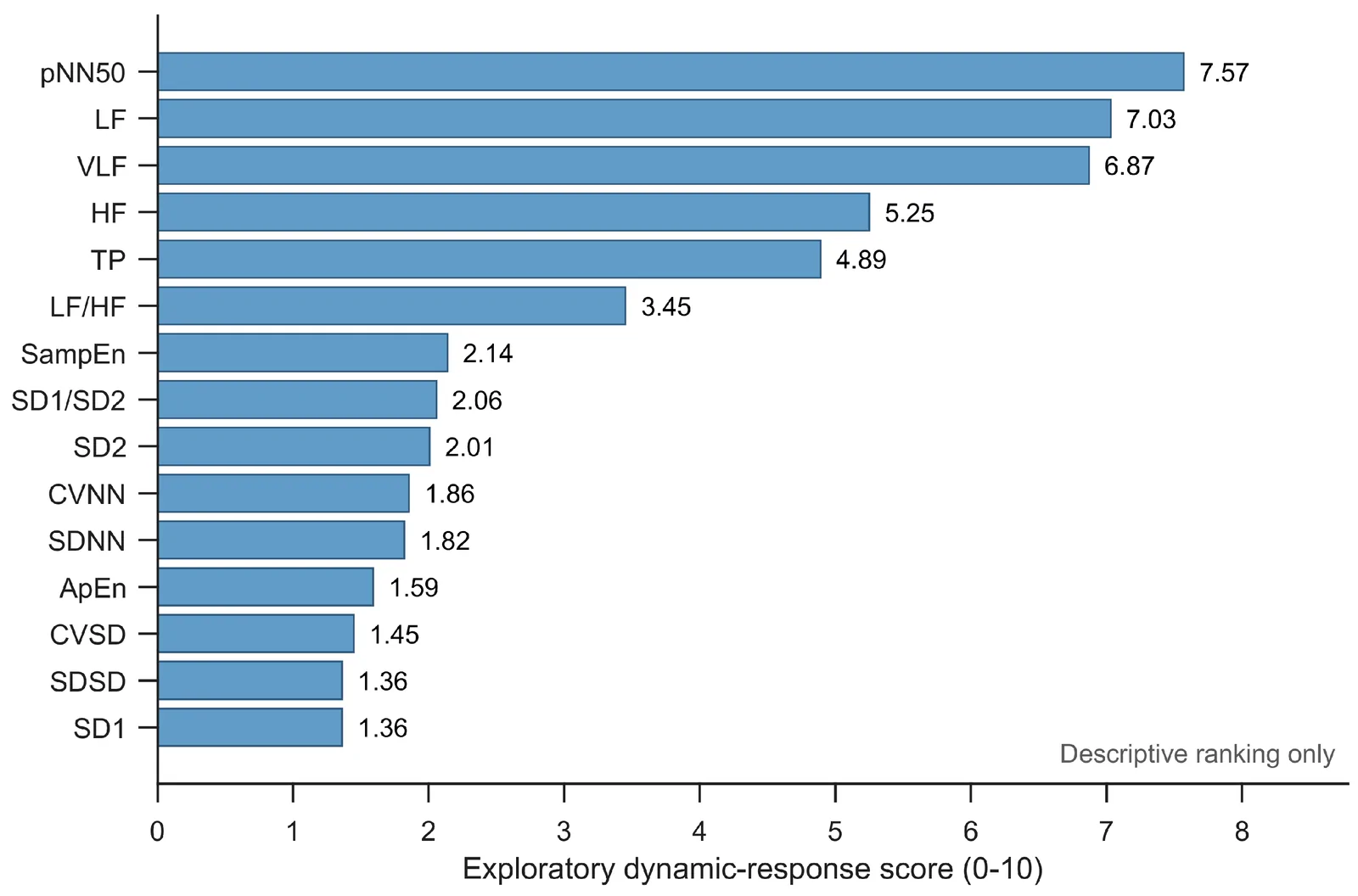
Exploratory dynamic-response ranking under the reference weighting scheme. The ranking is descriptive and was not used to select features for confirmatory testing.

**Figure 6:**
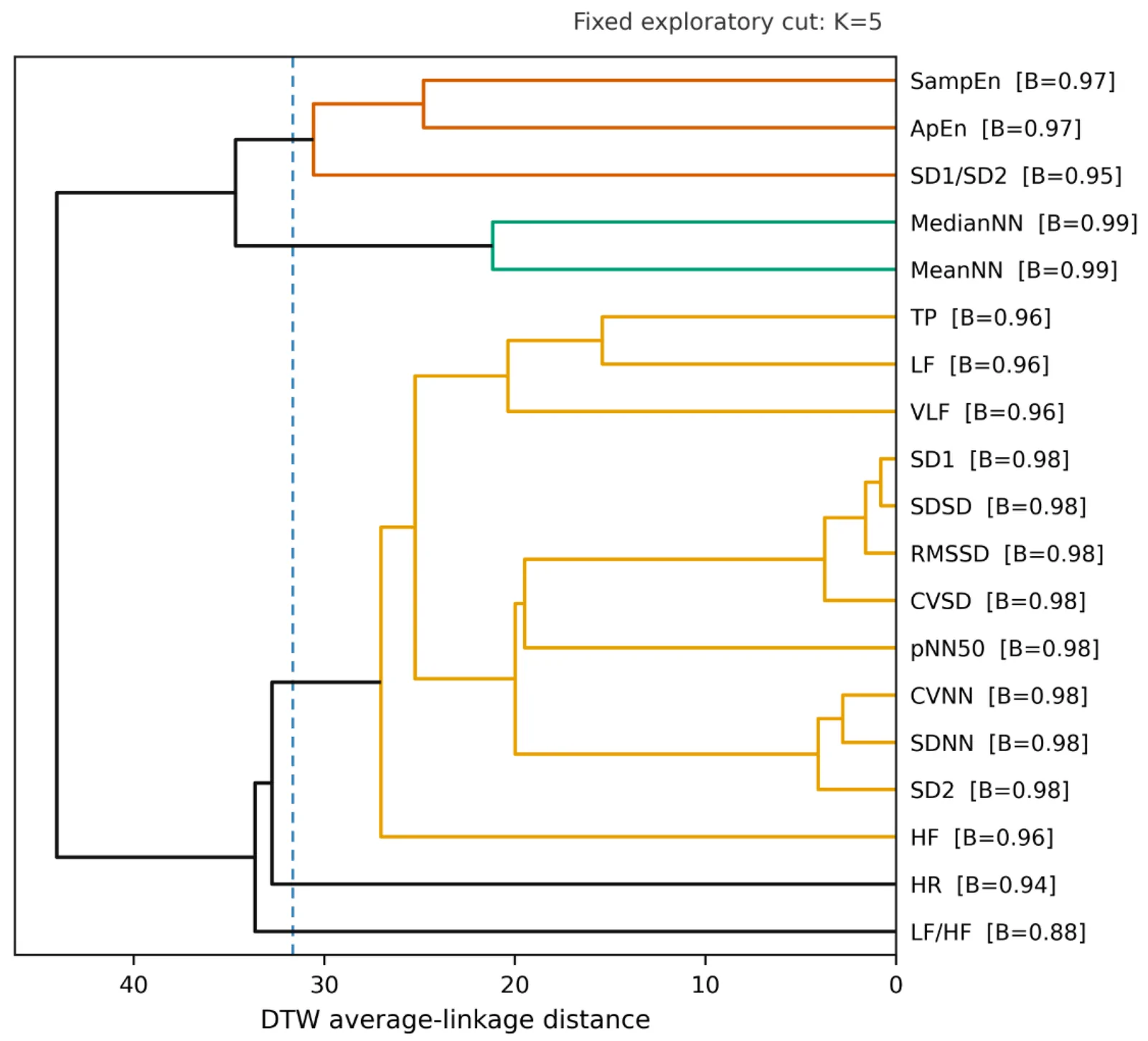
Average-linkage dendrogram based on DTW distances between z-standardized group-mean HRV feature trajectories. The dashed line shows the fixed exploratory K=5 cut. B values in the feature labels denote the implemented participant-bootstrap stability measure. The five-cluster cut is not presented as the optimal solution.

**Table 1:** Participant characteristics and analysis flow.

Characteristic	Value
Enrolled and completed pre–post manipulation checks	26
Sex	16 women; 10 men
Age, years	25±2
Body mass index, kg/m^2^	20.97±2.00
Included in HRV analysis	22
Excluded from HRV analysis	4; insufficient protocol compliance leading to inadequate valid signal duration, RR intervals, or windows
A priori sample-size calculation	Not performed; exploratory study

**Table 2:** Summary of temporal metrics and their analytical roles.

Metric	Definition	Interpretation	Role
Delta	$\left(F_{i}-F_{\mathrm{base}}\right)/F_{\mathrm{base}}$	Dimensionless cumulative deviation from baseline	Descriptive trajectory
LCR	$\ln\left(F_{i}+\varepsilon \right)-\ln\left(F_{i-1}+\varepsilon \right)$, $\varepsilon =10^{-6}$	Local proportional change between adjacent windows	Primary dynamic metric
CR%	$100\left(F_{i}-F_{i-1}\right)/F_{i-1}$	Conventional adjacent-window percentage change	Auxiliary only
Median(|LCR|)	Median absolute LCR within a phase	Participant-level phase change intensity	Friedman outcome
ZC	Number of sign changes in LCR	Directional-switching frequency	Exploratory morphology metric
Exploratory dynamic-response score	Weighted normalized combination of trend, phase spread, |LCR| strength, and ZC	Dataset-specific descriptive ranking	Not used for confirmatory selection

**Table 3:** Pre–post fatigue-manipulation checks (n=26).

Outcome	Pre, Mean ± SD	Post, Mean ± SD	Mean Change (95% CI)	Paired *t*	*p*	Wilcoxon *W*; *p*	Cohen’s $d_{z}$
FSAS mental- fatigue score	18.99±17.09	46.88±24.89	27.88 (19.34, 36.43)	t(25)=6.72	<0.001	0; <0.001	1.32
Reaction time, ms	371.63±37.74	407.62±66.70	35.98 (21.17, 50.80)	t(25)=5.00	<0.001	11; <0.001	0.98

**Table 4:** Descriptive respiratory-surrogate phase tests (n=22).

Metric	*n*	$\chi^{2}$	df	Raw *p*	Kendall’s *W*	FDR *p*
Respiratory rate (breaths/min)	22	3.482	3	0.3231	0.053	0.3231
Relative respiratory amplitude (ratio)	22	13.745	3	0.0033	0.208	0.0065

**Table 5:** Friedman phase tests for all 19 HRV features included in the primary analysis.

Feature	*n*	$\chi^{2}$	df	Raw *p*	Kendall’s *W*	BH-FDR *p*	FDR Significant
CVSD	22	13.200	3	0.0042	0.200	0.0596	No
RMSSD	22	11.018	3	0.0116	0.167	0.0596	No
SDSD	22	10.855	3	0.0125	0.164	0.0596	No
SD1	22	10.855	3	0.0125	0.164	0.0596	No
VLF	22	10.309	3	0.0161	0.156	0.0612	No
pNN50	22	8.564	3	0.0357	0.130	0.1130	No
MedianNN	22	5.291	3	0.1517	0.080	0.3603	No
SD1/SD2	22	5.291	3	0.1517	0.080	0.3603	No
ApEn	22	4.309	3	0.2300	0.065	0.4855	No
TP	22	2.727	3	0.4356	0.041	0.8013	No
HF	22	2.564	3	0.4639	0.039	0.8013	No
SampEn	22	2.073	3	0.5574	0.031	0.8826	No
MeanNN	22	1.527	3	0.6760	0.023	0.9098	No
HR	22	1.527	3	0.6760	0.023	0.9098	No
CVNN	22	1.255	3	0.7400	0.019	0.9098	No
LF/HF	22	1.145	3	0.7661	0.017	0.9098	No
LF	22	0.709	3	0.8711	0.011	0.9209	No
SDNN	22	0.655	3	0.8838	0.010	0.9209	No
SD2	22	0.491	3	0.9209	0.007	0.9209	No

**Table 6:** Exploratory dynamic-response ranking and random-weight uncertainty.

Feature	Ref. Score	Ref. Rank	Median RW Rank	Range	P (Top 5)
pNN50	7.57	1	2	1–12	81.6%
LF	7.03	2	2	1–6	99.6%
VLF	6.87	3	2	1–7	97.2%
HF	5.25	4	4	3–9	83.1%
TP	4.89	5	5	3–16	59.2%
LF/HF	3.45	6	6	1–13	49.2%
SampEn	2.14	7	7	1–15	18.8%
SD1/SD2	2.06	8	8	3–15	8.9%

**Table 7:** Fixed five-cluster exploratory DTW solution.

Cluster	Size	Features	Mean Silhouette	Bootstrap Stability Range
1	1	HR	0.000	0.941–0.941
2	12	CVNN, CVSD, HF, LF, RMSSD, SD1, SD2, SDNN, SDSD, TP, VLF, pNN50	0.338	0.956–0.975
3	1	LF/HF	0.000	0.875–0.875
4	2	MeanNN, MedianNN	0.389	0.989–0.989
5	3	ApEn, SD1/SD2, SampEn	0.174	0.948–0.970

**Table 8:** Summary of segmentation and LCR parameter sensitivity analyses.

Analysis	Setting	Result
Alternative segmentation	4×15 min	0 FDR-significant features; *W*-ranking ρ=0.772 vs. primary
Alternative segmentation	3×20 min	0 FDR-significant features; *W*-ranking ρ=0.830 vs. primary
Window/step	3 min/1 min	1 FDR-significant feature
Window/step	5 min/1 min	0 FDR-significant features; primary
Window/step	5 min/2 min	0 FDR-significant features
Window/step	5 min/5 min	11 FDR-significant features
Epsilon	$10^{-8}$, $10^{-6}$, $10^{-4}$	No within-configuration change in conclusions

## Data Availability

The data presented in this study are available from the corresponding author upon reasonable request. The data are not publicly available because of ethical and privacy restrictions related to human-participant physiological recordings.

## Supplementary Materials

The following supporting information can be downloaded at https://www.mdpi.com/article/10.3390/s26196099/s1, Table S1. Phase summaries of participant-level median |LCR|; Table S2. Fixed K = 5 feature-level cluster results; Table S3. Correlation of each participant trajectory with the leave-one-participant-out group trajectory; Figure S1. Descriptive BCG-derived respiratory-surrogate results. Relative amplitude is uncalibrated and should not be interpreted as respiratory depth; Figure S2. Sensitivity of feature-level phase effect sizes to alternative segmentation schemes; Figure S3. Exploratory rank uncertainty under 5000 randomly sampled weight vectors; Figure S4. Exploratory Delta trajectories for top-ranked features. Delta is a dimensionless ratio; shaded bands are t-based 95% confidence intervals around the group mean.

## Abbreviations

ApEn	Approximate entropy
BCG	Ballistocardiogram
CR%	Percentage change between adjacent windows
CVNN	Coefficient of variation of NN intervals
CVSD	Coefficient of variation of successive differences
DTW	Dynamic time warping
ECG	Electrocardiogram
FDR	False discovery rate
FSAS	Fatigue Self-Assessment Scale
HF	High frequency
HR	Heart rate
HRV	Heart-rate variability
JJ	Interval between successive BCG J peaks
LCR	Log change rate
LF	Low frequency
MZI	Mach–Zehnder interferometer
NN	Normal-to-normal interval
pNN50	Percentage of adjacent NN intervals differing by more than 50 ms
RMSSD	Root mean square of successive differences
RR	Interval between successive ECG R peaks
SampEn	Sample entropy
SDNN	Standard deviation of NN intervals
SDSD	Standard deviation of successive differences
TP	Total power
VLF	Very low frequency
ZC	Zero-crossing count
