# Supplementary material for: Exploratory Temporal Dynamics of Heart-Rate Variability During a Prolonged 2-Back Task Under Nap-Deprivation Conditions
Source: Sensors (Basel). 2026 Sep 25;26(19):6099. doi: 10.3390/s26196099 (PMC13667429; doi:10.3390/s26196099)
Supplement: Supplementary file 1 [file sensors-26-06099-s001.zip › sensors-4518525-supplementary.pdf]

Supplementary Material

for "Exploratory Temporal Dynamics of Heart Rate Variability During a Prolonged 2-Back Task Under Nap-Deprivation Conditions"

Table S1. Phase summaries of participant-level median |LCR|.

Feature	Phase	n	Median	Q1	Q3	Mean	SD
MeanNN	Early (0-10)	22	0.00624	0.00495	0.00967	0.00739	0.00419
MeanNN	Change (10-20)	22	0.00532	0.00360	0.00821	0.00664	0.00592
MeanNN	Mid (20-40)	22	0.00664	0.00406	0.00846	0.00671	0.00329
MeanNN	Late (40-60)	22	0.00696	0.00453	0.00897	0.00740	0.00390
MedianNN	Early (0-10)	22	0.00624	0.00401	0.00914	0.00735	0.00426
MedianNN	Change (10-20)	22	0.00429	0.00303	0.00664	0.00535	0.00434
MedianNN	Mid (20-40)	22	0.00519	0.00358	0.00653	0.00538	0.00197
MedianNN	Late (40-60)	22	0.00577	0.00345	0.00823	0.00583	0.00246
SDNN	Early (0-10)	22	0.05551	0.03028	0.06872	0.05693	0.02951
SDNN	Change (10-20)	22	0.05637	0.04130	0.08099	0.06318	0.03301
SDNN	Mid (20-40)	22	0.05556	0.04751	0.07068	0.05869	0.01788
SDNN	Late (40-60)	22	0.05399	0.03744	0.07145	0.05946	0.02776
RMSSD	Early (0-10)	22	0.06545	0.05601	0.08675	0.07074	0.01985
RMSSD	Change (10-20)	22	0.05590	0.02921	0.09164	0.06307	0.03755
RMSSD	Mid (20-40)	22	0.04760	0.03930	0.05389	0.05117	0.02145
RMSSD	Late (40-60)	22	0.04998	0.03990	0.06162	0.05471	0.02209
SDSD	Early (0-10)	22	0.06546	0.05609	0.08694	0.07080	0.01988
SDSD	Change (10-20)	22	0.05591	0.02917	0.09166	0.06309	0.03761
SDSD	Mid (20-40)	22	0.04761	0.03931	0.05391	0.05120	0.02150
SDSD	Late (40-60)	22	0.05001	0.03989	0.06171	0.05472	0.02212
pNN50	Early (0-10)	22	0.14267	0.11304	0.16985	0.14089	0.05453
pNN50	Change (10-20)	22	0.10098	0.07488	0.14477	0.12886	0.07316
pNN50	Mid (20-40)	22	0.09850	0.07601	0.11994	0.10090	0.03704
pNN50	Late (40-60)	22	0.07696	0.06171	0.10276	0.09307	0.04736
CVNN	Early (0-10)	22	0.05766	0.03485	0.06590	0.05916	0.02948
CVNN	Change (10-20)	22	0.05512	0.04017	0.07176	0.06127	0.03365
CVNN	Mid (20-40)	22	0.06051	0.04673	0.07363	0.06079	0.01863
CVNN	Late (40-60)	22	0.05420	0.03647	0.06969	0.05779	0.02711

Feature	Phase	n	Median	Q1	Q3	Mean	SD
CVSD	Early (0-10)	22	0.07195	0.05929	0.08709	0.07095	0.02054
CVSD	Change (10-20)	22	0.05488	0.03247	0.09229	0.06089	0.03359
CVSD	Mid (20-40)	22	0.05000	0.03698	0.05895	0.05022	0.01978
CVSD	Late (40-60)	22	0.04923	0.04002	0.06723	0.05507	0.02093
HR	Early (0-10)	22	0.00624	0.00495	0.00967	0.00739	0.00419
HR	Change (10-20)	22	0.00532	0.00360	0.00821	0.00664	0.00592
HR	Mid (20-40)	22	0.00664	0.00406	0.00846	0.00671	0.00329
HR	Late (40-60)	22	0.00696	0.00453	0.00897	0.00740	0.00390
VLF	Early (0-10)	22	0.27603	0.20871	0.35507	0.29009	0.10967
VLF	Change (10-20)	22	0.23947	0.16449	0.32580	0.24762	0.11504
VLF	Mid (20-40)	22	0.22001	0.17911	0.30549	0.25640	0.12915
VLF	Late (40-60)	22	0.18732	0.15705	0.23961	0.20827	0.06547
LF	Early (0-10)	22	0.18861	0.13345	0.21995	0.18831	0.06827
LF	Change (10-20)	22	0.17721	0.12932	0.26615	0.21120	0.11194
LF	Mid (20-40)	22	0.17659	0.14076	0.21115	0.19525	0.08094
LF	Late (40-60)	22	0.17267	0.13707	0.20694	0.18268	0.07042
HF	Early (0-10)	22	0.17568	0.09885	0.22105	0.16285	0.06603
HF	Change (10-20)	22	0.17002	0.11378	0.21655	0.17787	0.07230
HF	Mid (20-40)	22	0.11295	0.09290	0.17576	0.13593	0.07510
HF	Late (40-60)	22	0.14896	0.09816	0.17518	0.14957	0.06360
LF/HF	Early (0-10)	22	0.17274	0.13613	0.19931	0.18172	0.07011
LF/HF	Change (10-20)	22	0.17146	0.12797	0.20162	0.16739	0.05012
LF/HF	Mid (20-40)	22	0.14512	0.12363	0.16706	0.14993	0.04031
LF/HF	Late (40-60)	22	0.14660	0.12982	0.19467	0.16176	0.04853
TP	Early (0-10)	22	0.18464	0.12386	0.22934	0.18877	0.07805
TP	Change (10-20)	22	0.17023	0.11240	0.24449	0.18433	0.10176
TP	Mid (20-40)	22	0.15475	0.10310	0.21178	0.18148	0.11193
TP	Late (40-60)	22	0.14247	0.12202	0.17643	0.15555	0.06487
SD1	Early (0-10)	22	0.06546	0.05609	0.08694	0.07080	0.01988
SD1	Change (10-20)	22	0.05591	0.02917	0.09166	0.06309	0.03761
SD1	Mid (20-40)	22	0.04761	0.03931	0.05391	0.05120	0.02150

Feature	Phase	n	Median	Q1	Q3	Mean	SD
SD1	Late (40-60)	22	0.05001	0.03989	0.06171	0.05472	0.02212
SD2	Early (0-10)	22	0.05161	0.04077	0.07115	0.06205	0.03531
SD2	Change (10-20)	22	0.05944	0.04553	0.08211	0.06632	0.03390
SD2	Mid (20-40)	22	0.05918	0.05218	0.07270	0.06293	0.01805
SD2	Late (40-60)	22	0.06017	0.04156	0.07552	0.06468	0.02985
SD1/SD2	Early (0-10)	22	0.07292	0.05604	0.09984	0.07767	0.03652
SD1/SD2	Change (10-20)	22	0.05847	0.03726	0.07409	0.06134	0.02915
SD1/SD2	Mid (20-40)	22	0.05717	0.04775	0.07056	0.05970	0.01783
SD1/SD2	Late (40-60)	22	0.05519	0.04923	0.06492	0.05843	0.01633
ApEn	Early (0-10)	22	0.03284	0.02080	0.04109	0.03873	0.03381
ApEn	Change (10-20)	22	0.02901	0.01918	0.03928	0.03096	0.01445
ApEn	Mid (20-40)	22	0.02760	0.01601	0.03316	0.02707	0.01372
ApEn	Late (40-60)	22	0.02897	0.01875	0.03760	0.03134	0.01560
SampEn	Early (0-10)	22	0.05314	0.04662	0.06967	0.06495	0.04117
SampEn	Change (10-20)	22	0.04942	0.03235	0.07077	0.05557	0.03132
SampEn	Mid (20-40)	22	0.05132	0.04211	0.06432	0.05483	0.01765
SampEn	Late (40-60)	22	0.05276	0.03468	0.07554	0.05754	0.02609

Table S2. Fixed K=5 feature-level cluster results.

Feature	Cluster	Cluster size	Silhouette	Bootstrap stability
HR	1	1	0.000	0.941
CVNN	2	12	0.417	0.975
CVSD	2	12	0.411	0.975
HF	2	12	0.185	0.961
LF	2	12	0.038	0.956
RMSSD	2	12	0.435	0.975
SD1	2	12	0.435	0.975
SD2	2	12	0.392	0.975
SDNN	2	12	0.432	0.975
SDSD	2	12	0.435	0.975
TP	2	12	0.273	0.957

Feature	Cluster	Cluster size	Silhouette	Bootstrap stability
VLF	2	12	0.260	0.956
pNN50	2	12	0.348	0.975
LF/HF	3	1	0.000	0.875
MeanNN	4	2	0.397	0.989
MedianNN	4	2	0.381	0.989
ApEn	5	3	0.192	0.968
SD1/SD2	5	3	0.129	0.948
SampEn	5	3	0.200	0.970

Table S3. Correlation of each participant trajectory with the leave-one-participant-out group trajectory.

Feature	n	Median rho	Q1	Q3	Positive	rho >= 0.30
MeanNN	22	0.033	-0.061	0.142	54.5%	0.0%
MedianNN	22	-0.035	-0.125	0.076	45.5%	4.5%
SDNN	22	0.093	0.002	0.186	77.3%	9.1%
RMSSD	22	0.069	-0.001	0.159	72.7%	0.0%
SDSD	22	0.069	-0.001	0.159	72.7%	0.0%
pNN50	22	0.115	0.028	0.166	90.9%	4.5%
CVNN	22	0.079	0.010	0.219	86.4%	4.5%
CVSD	22	0.072	0.006	0.158	77.3%	0.0%
HR	22	0.033	-0.061	0.142	54.5%	0.0%
VLF	22	-0.051	-0.147	0.044	36.4%	0.0%
LF	22	0.081	-0.012	0.159	72.7%	4.5%
HF	22	0.014	-0.053	0.142	59.1%	0.0%
LF/HF	22	0.075	-0.081	0.226	54.5%	9.1%
TP	22	-0.006	-0.089	0.115	50.0%	4.5%
SD1	22	0.069	-0.001	0.159	72.7%	0.0%
SD2	22	0.080	-0.006	0.206	72.7%	4.5%
SD1/SD2	22	-0.091	-0.146	0.137	36.4%	0.0%
ApEn	22	0.052	-0.066	0.157	54.5%	4.5%
SampEn	22	0.068	-0.055	0.133	59.1%	9.1%

Supplementary Figures

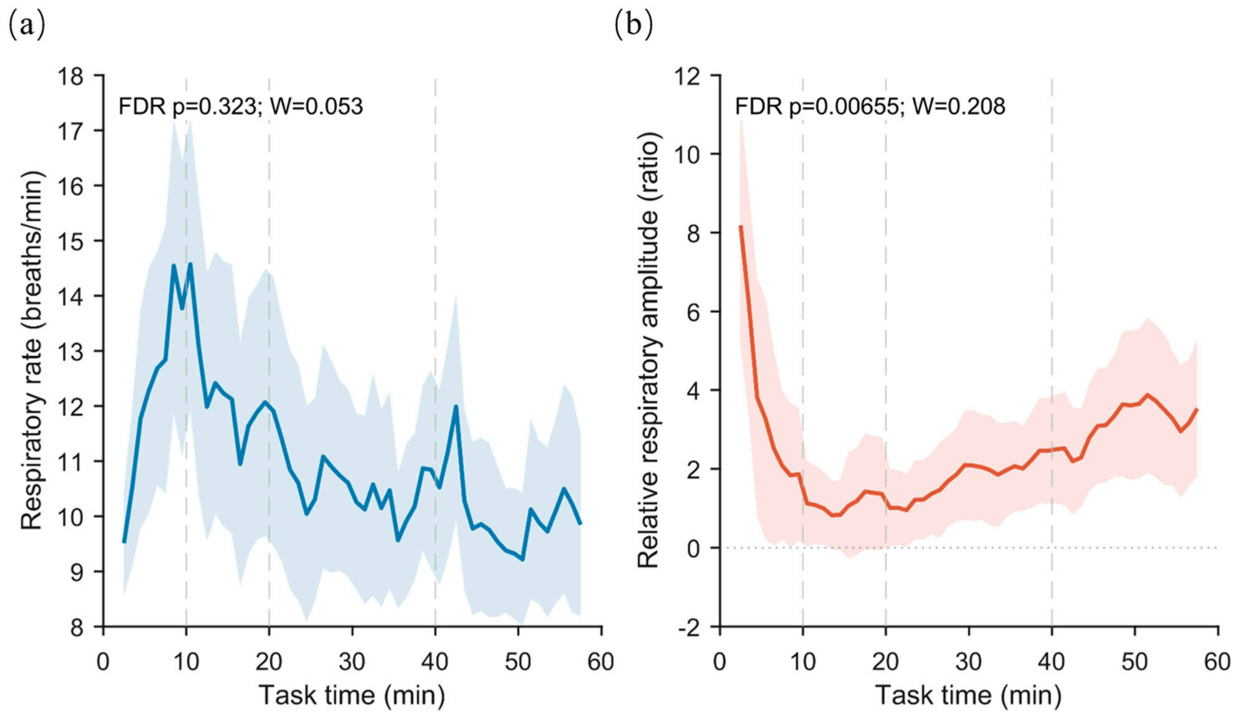

Figure S1. Descriptive BCG-derived respiratory-surrogate results. Relative amplitude is uncalibrated and should not be interpreted as respiratory depth.

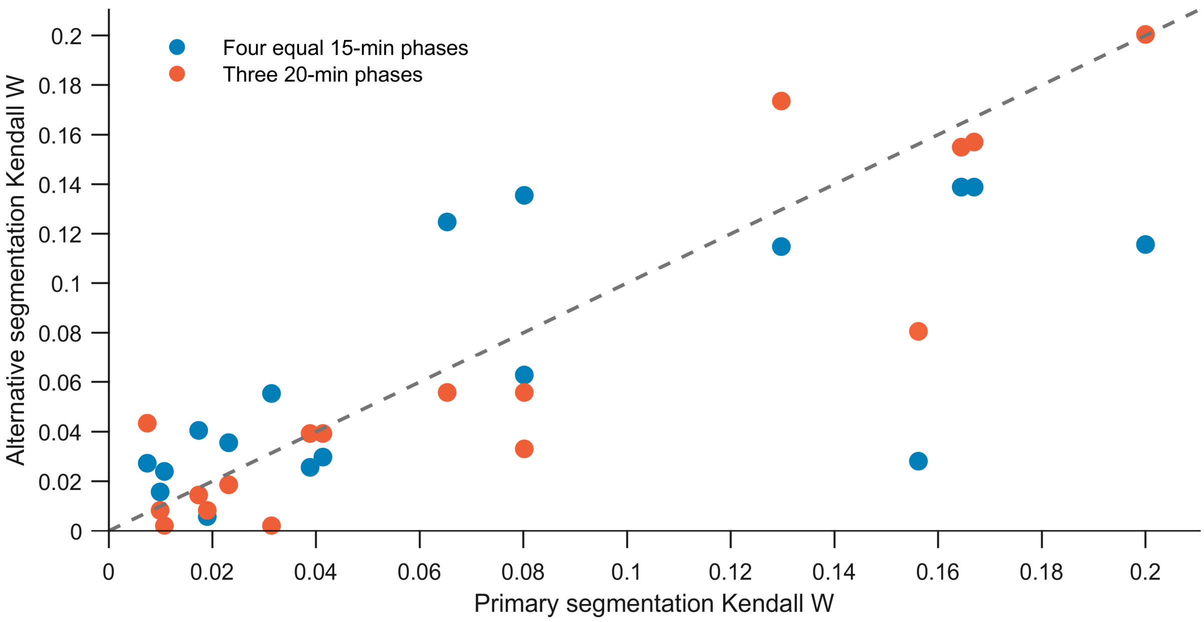

Figure S2. Sensitivity of feature-level phase effect sizes to alternative segmentation schemes.

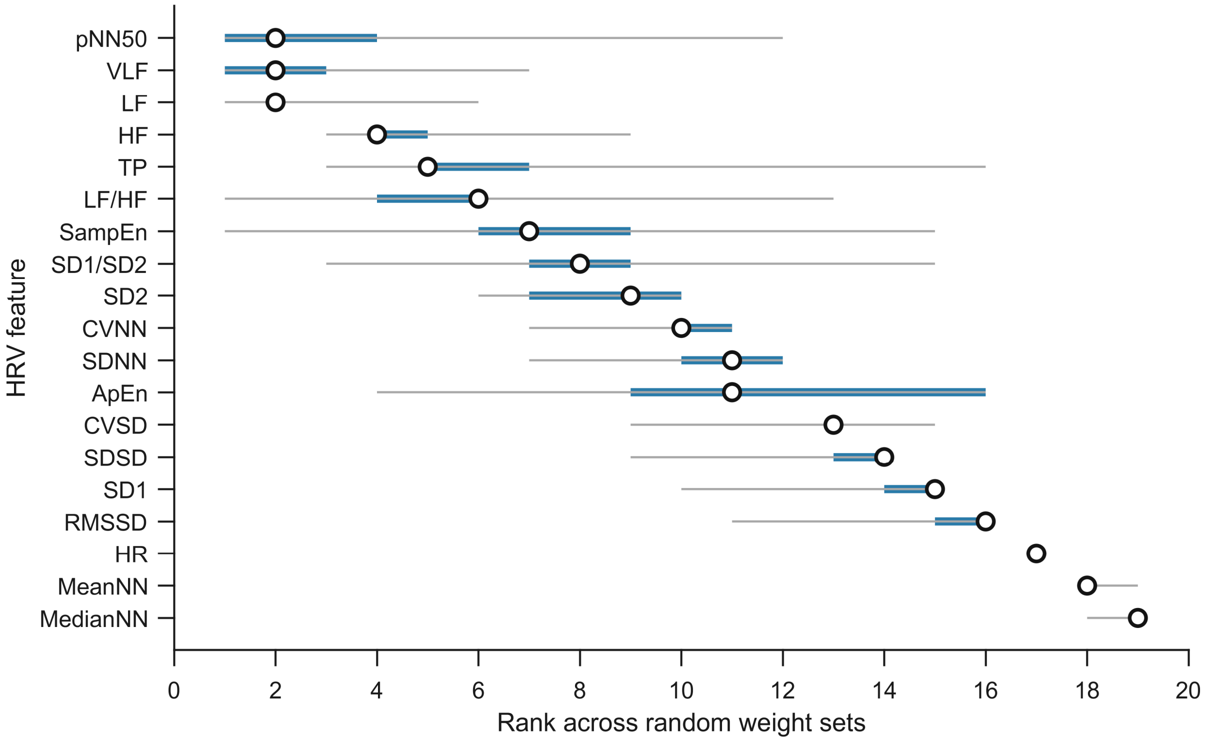

Figure S3. Exploratory rank uncertainty under 5000 randomly sampled weight vectors.

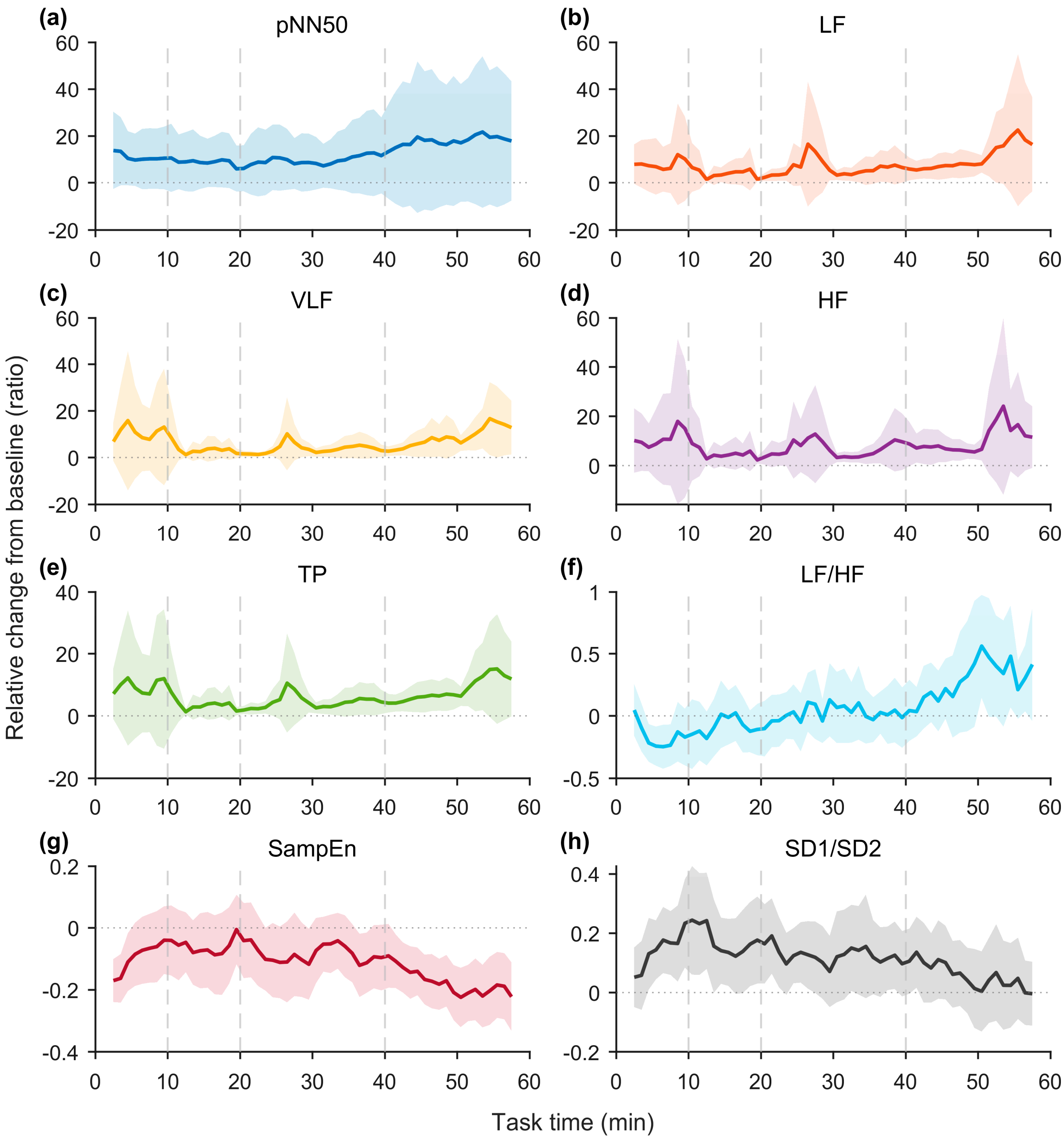

Figure S4. Exploratory Delta trajectories for top-ranked features. Delta is a dimensionless ratio; shaded bands are t-based 95% confidence intervals around the group mean.
